# Romidepsin for the Treatment of Peripheral T-Cell Lymphoma

**Published:** 2015-01-01

**Authors:** Lisa Barbarotta, Kristen Hurley

**Affiliations:** 1Hematology-Oncology Service, Smilow Cancer Hospital, Yale New Haven, Connecticut; 2Avera Medical Group, Hematology and Bone Marrow Transplantation, Sioux Falls, South Dakota

## Abstract

Peripheral T-cell lymphomas (PTCLs) are a rare, heterogeneous group of T-cell– or natural killer cell–derived non-Hodgkin lymphomas. The majority of patients with PTCL experience an aggressive disease course and poor overall survival. Historically, PTCL has been treated with chemotherapy regimens used to treat B-cell lymphomas; however, a lack of durable responses to frontline therapies and few effective options for salvage treatment have led to the development of newer therapies. Romidepsin is a structurally unique, potent, bicyclic class 1 selective histone deacetylase (HDAC) inhibitor that has demonstrated durable clinical responses in patients with relapsed/refractory PTCL, leading to its approval by the US Food and Drug Administration in 2011 for the treatment of PTCL in patients who have received at least one prior therapy. Here, the authors provide an overview of PTCL, review the role of HDAC inhibitors as anticancer agents, discuss romidepsin use in PTCL, and highlight considerations for advanced practitioners (including the management of side effects).

Peripheral T-cell lymphomas (PTCLs) are a heterogeneous group of aggressive, uncommon forms of T-cell– or natural killer (NK)–cell-derived non-Hodgkin lymphomas (NHLs) that are typically associated with a poor prognosis (Foss et al., 2011; Horwitz, 2007; Vose, Armitage, Weisenburger, & International T-Cell Lymphoma Project, 2008). The term "peripheral" refers not to anatomic site but to the fact that PTCL is derived from mature T cells (peripheral to the thymus; Horwitz, 2007). There are several subtypes of PTCL, distinguishable by immunophenotyping, molecular markers, and clinical signs, which have variable prognoses (Vose et al., 2008).

There is currently no standard of care for the treatment of PTCL and no approved agents for first-line treatment (NCCN, 2014). Anthracycline-containing regimens such as CHOP (cyclophosphamide, doxorubicin, vincristine, prednisone) are commonly used in patients with newly diagnosed PTCL; however, the majority of patients do not experience durable responses or long-term disease-free survival (Vose et al., 2008). Furthermore, when these treatments fail, there are few effective options for salvage therapy (Foss et al., 2011). The role of autologous stem cell transplantation (ASCT) is still under examination; retrospective studies have demonstrated that some patients with PTCL may achieve benefit from ASCT (Foss et al., 2011; Yared & Kimball, 2013), but < 15% of patients with T-cell lymphoma currently receive ASCT (Evens et al., 2012; Foss et al., 2012b).

New agents have been under investigation to try to improve outcomes in patients with PTCL. Romidepsin (Istodax), a histone deacetylase (HDAC) inhibitor, was approved by the US Food and Drug Administration (FDA) in November 2009 for the treatment of cutaneous T-cell lymphoma (CTCL) in patients who have received at least one prior systemic therapy and in May 2011 for the treatment of PTCL in patients who have received at least one prior therapy (Celgene Corporation, 2014).

This review will provide an overview of PTCL, including its diagnosis and treatment; discuss the role of HDAC inhibitors as anticancer agents; describe romidepsin and its use in clinical trials for patients with relapsed/refractory PTCL; and summarize recommendations for advanced practitioners (APs) when caring for patients receiving romidepsin for PTCL.

## PERIPHERAL T-CELL LYMPHOMA

Non-Hodgkin lymphoma is a diverse group of lymphoproliferative cancers, of which PTCL accounts for approximately 10% of all cases (NCCN, 2014). PTCL is a heterogeneous group of uncommon, mature, post-thymic, T- and NK-cell disorders (Horwitz, 2007). Over the past few decades, researchers have proposed a number of different classification systems for T- and NK-cell lymphomas (Foss et al., 2011). The most recent classification by the World Health Organization in 2008 listed the distinct subtypes of PTCL, expanding some previous subtypes and adding several new provisional diseases (Table 1; Vose et al., 2008). PTCL is distinct from CTCL, another rare form of NHL that arises from the skin (Leukemia & Lymphoma Society, 2012; NCCN, 2014). The presentation, treatment, and disease course of CTCL are different from PTCL and will not be discussed here.

**Table 1:**
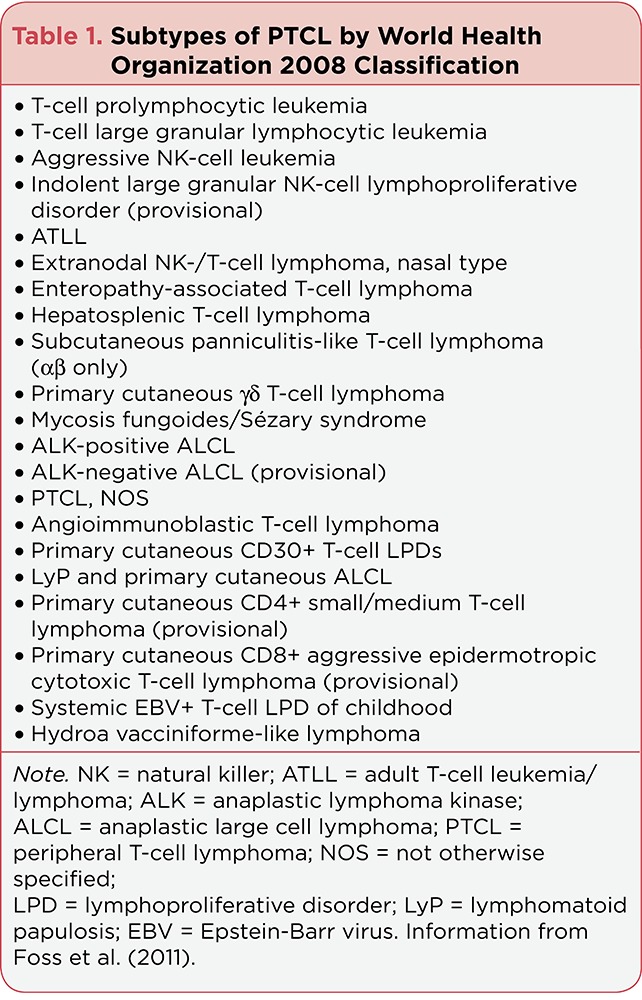
Subtypes of PTCL by World Health Organization 2008 Classification

**Diagnosis and Staging**

Because PTCL is a heterogeneous group of diseases, clinical features also vary widely (Leukemia & Lymphoma Society, 2012). Common symptoms include fatigue, weight loss, rash, enlarged lymph nodes, and night sweats (Leukemia & Lymphoma Society, 2012). Furthermore, a number of organs may be affected, including the bone marrow, liver, spleen, skin, and stomach (Leukemia & Lymphoma Society, 2012).

A diagnosis of PTCL occurs first by excisional lymph node biopsy; fine-needle aspiration alone is not sufficient (NCCN, 2014). To ensure accurate diagnosis, other ancillary techniques such as immunohistochemistry and flow cytometry should be used (NCCN, 2014). Cells are assessed to determine whether they express B-, T-, or NK-cell markers; T- or NK-cell lymphomas express one or more T- or NK-/T-cell antigens (CD2, CD3, CD5, CD7) but not B-cell antigens (CD19, CD20, CD79a, PAX5; NCCN, 2014). T-cell antigen-positive immunophenotype samples are then differentiated by morphology and location (NCCN, 2014).

Cell morphology is determined, based on size and shape, including whether or not cells exhibit anaplastic morphology (NCCN, 2014). The location of the lesion (nodal vs. extranodal, cutaneous vs. noncutaneous) and other clinical data, such as the age of the patient, are also considered (NCCN, 2014). A series of specific immunophenotypic panels are used to establish a subtype diagnosis by determining the antigens expressed as well as human T-lymphotropic virus-1 (HTLV-1) status (NCCN, 2014). The full algorithm for determining PTCL subtype by immunophenotyping can be found in the NCCN guidelines for NHL (NCCN, 2014). Proper diagnosis requires review by an expert hematopathologist (Vose et al., 2008).

Staging of PTCL involves a complete physical examination plus routine laboratory tests, such as a complete blood cell count, renal and liver function tests, serum lactate dehydrogenase (LDH) measurement, bone marrow examination, and radiologic imaging (NCCN, 2014; Tang et al., 2010). Computed tomography (CT) and/or fluoro-deoxyglucose positron emission tomography (FDG-PET)/CT are essential for baseline staging and follow-up examinations (NCCN, 2014; Tang et al., 2010).

On the basis of the results of these tests, patients are staged using the Ann Arbor staging system (Armitage, 2012), originally created for Hodgkin lymphoma (Carbone, Kaplan, Musshoff, Smithers, & Tubiana, 1971). Stage I disease involves a single lymph node/lymph node region or single extranodal site. Stage II disease involves two or more lymph node regions on the same side of the diaphragm or an extranodal site plus lymph involvement on the same side of the diaphragm. Stage III disease involves lymph node regions on both sides of the diaphragm, with or without partial involvement of an extranodal organ or site. Lastly, stage IV disease demonstrates diffuse or disseminated involvement, including involvement in one or more extranodal sites, with or without lymph node enlargement (Carbone et al., 1971; Leukemia & Lymphoma Society, 2012). Unfortunately, the majority of patients with PTCL present with advanced-stage disease (Armitage, 2012; Vose et al., 2008).

**Epidemiology**

Non-Hodgkin lymphoma accounts for approximately 4% of all cancers diagnosed in the United States (American Cancer Society, 2014; National Cancer Institute, 2014). The US prevalence of NHL in 2011 was estimated to be 530,919 people, affecting a slightly greater number of men than women (National Cancer Institute, 2014). Peripheral T-cell lymphoma accounts for approximately 10% of the estimated 70,800 new cases of NHL diagnosed yearly in the United States (American Cancer Society, 2014; National Cancer Institute, 2014; Horwitz, 2007), and the median age at diagnosis is 62 years (Vose et al., 2008). The frequency of T- and NK-cell lymphomas has been found to vary geographically, with the highest incidence in Asia (Anderson, Armitage, & Weisenburger, 1998; Armitage, 2012; Ascani et al., 1997; Nakamura et al., 1993; Vose et al., 2008).

With the exception of anaplastic lymphoma kinase (ALK)–positive anaplastic large cell lymphoma (ALCL), the incidence of PTCL is increasing and has more than tripled since 1992 (Petrich, Helenowski, Galamaga, & Nabhan, 2012), which is attributed to improvements in the accuracy of diagnosis and to an aging population (Lymphoma Research Foundation, 2013). However, overall survival (OS) has not increased and, in fact, suggests deterioration in outcomes over time (Petrich et al., 2012).

**Figure 1:**
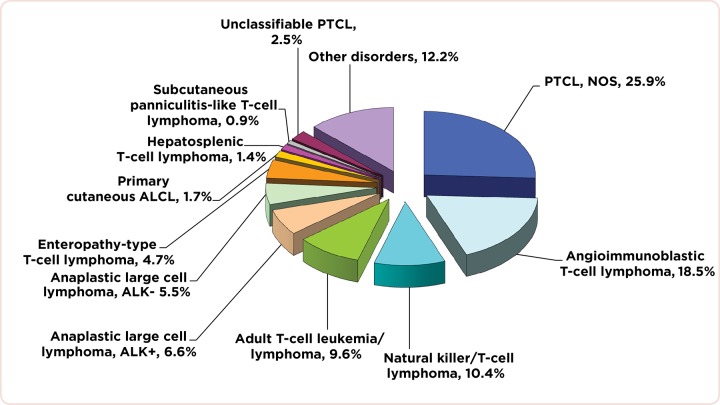
Figure 1. Global distribution of 1,314 cases of PTCL by consensus diagnosis. Other disorders are cases that were misclassified as PTCL (10.4%) and cases with other T-cell disorders not specifically included in the study (1.8%). ALCL = anaplastic large cell lymphoma; ALK = anaplastic lymphoma kinase; NOS = not otherwise specified; PTCL = peripheral T-cell lymphoma. Adapted with permission from Vose et al. (2008). Reprinted with permission. © 2008 American Society of Clinical Oncology. All rights reserved.

The International Peripheral T-Cell and Natural Killer/T-Cell Lymphoma Study from North America, Europe, and Asia found that the two most prevalent subtypes were PTCL, not otherwise specified (NOS) and angioimmunoblastic T-cell lymphoma (AITL; Figure 1; Vose et al., 2008). This study also found that the relative frequency of PTCL subtypes varies by geographic region; for example, the frequency of AITL was highest in Europe, and the frequencies of NK/T-cell lymphoma (NKTCL) and adult T-cell leukemia/lymphoma (ATLL) were highest in Asia. Some of this variation may be a result of exposure or genetic susceptibility to pathogenic agents, such as HTLV-1 and Epstein-Barr virus (EBV) in Asia (Anderson et al., 1998; Jaffe et al., 1996; Mahieux & Gessain, 2003; Rüdiger et al., 2002; Vose et al., 2008). Recently, better characterization of the cellular origin and pathophysiology of PTCLs has led to the development of new diagnostic markers (Parrens et al., 2012). This has allowed for an amended diagnosis of some cases of PTCL, NOS to AITL (Parrens et al., 2012), and other cases of PTCL, NOS have been shown to have genomic aberrations resembling ATLL (Yoshida et al., 2012).

Among 1,314 cases reviewed in 2008, a diagnosis of PTCL or NKTCL was confirmed by expert hematopathologists in 1,153 of the cases (87.8%; Vose et al., 2008). Misclassification was common, occurring in 10.4% of cases (represented as part of "other disorders" in Figure 1; Vose et al., 2008). There were issues with reproducibility of subtype diagnoses made by expert pathologists, varying from 66% to 97% reproducibility by subtype. Subtypes with specific markers (e.g., ALK-positive ALCL) were more frequently agreed upon than were those without solid defining features (e.g., PTCL, NOS).

Another recent study examined 374 cases of PTCL for diagnostic accuracy and clinical relevance and found that experienced hematopathologists using a defined immunohistochemistry panel reached a consensus diagnosis in 93% of PTCL cases (across all subtypes examined; Hsi et al., 2012). However, diagnoses are not always made by expert hematopathologists. Diagnostic concordance between referring and NCCN centers for T-cell lymphomas occurred in only 39% of patients examined (Herrera et al., 2012). The majority of patients were referred to these centers with provisional diagnoses, many of which were discordant (Herrera et al., 2012). This finding highlights the need for an expert pathologic review to correctly diagnose PTCL.

**Etiology**

Only a few subtypes of PTCL have a known etiology, including those associated with viral infection (Jaffe et al., 1996; Mahieux & Gessain, 2003). HTLV-1, which infects 15 to 20 million people worldwide, is a known cause of ATLL (Mahieux & Gessain, 2003). Epstein-Barr virus also plays a causal role in the development of lymphoma and has been associated with extranodal NK-/T-cell lymphoma, nasal type (Jaffe et al., 1996). Other subtypes are frequently associated with particular translocations, although genetic features play little role in the definition of most subtypes (de Leval & Gaulard, 2011).

**Treatment**

With the exception of first-line treatment guidelines for ALK-positive ALCL, there is currently no standard of care for the treatment of PTCL and no approved agents for first-line treatment (NCCN, 2014). Anthracycline-containing regimens, such as CHOP, were initially used in T-cell lymphomas because of established success in B-cell lymphomas (Foss et al., 2011). Because a better alternative has not been found, these regimens are still commonly used, despite the fact that they frequently result in an inadequate response or a lack of durable remission (Vose et al., 2008).

Retrospective studies have shown that, in recent years, the majority of patients in the United States continue to receive CHOP or CHOP-like regimens (Evens et al., 2012; Foss et al., 2012). Anthracycline-containing regimens for PTCL have been associated with median 5-year OS rates of < 40% (Abouyabis, Shenoy, Flowers, & Lechowicz, 2007), despite a majority of patients experiencing complete response (CR) after first-line therapy (Biasoli et al., 2012; Foss et al., 2012). When chemotherapy fails, there are few effective treatments for salvage (Foss et al., 2011), and many patients die before receiving salvage therapy (Biasoli et al., 2012).

Because of these inferior outcomes, novel treatment strategies have been explored; however, because PTCL is a rare disease, development of optimal therapeutic approaches through clinical trials can be difficult (Foss et al., 2011; Horwitz, 2007). Approved agents for relapsed/refractory PTCL include HDAC inhibitors romidepsin and belinostat (Beleodaq), folate analog pralatrexate (Folotyn), and CD30-directed antibody-drug brentuximab vedotin (Adcetris, for ALCL; Celgene Corporation, 2013; Spectrum Pharmaceuticals, Inc., 2014; Allos Therapeutics, Inc., 2012; Seattle Genetics, Inc., 2013).

On the basis of trial results thus far, the NCCN has written detailed evidence-based treatment approaches for PTCL (NCCN, 2014). A clinical trial is preferred for both first- and second-line treatments, except for patients with ALK-positive ALCL. In the absence of suitable clinical trials, multiagent chemotherapy with or without radiotherapy is recommended. Retrospective studies on ASCT have led to the recommendation that all patients consider consolidation with ASCT in first remission, except for patients with ALK-positive ALCL in remission. Both newer agents and chemotherapy regimens are recommended for second-line treatment, and, when choosing therapy, consideration should be given to whether the patient is eligible for ASCT. Agents recommended for first- and second-line therapies are listed in Table 2 (NCCN, 2014).

**Table 2:**
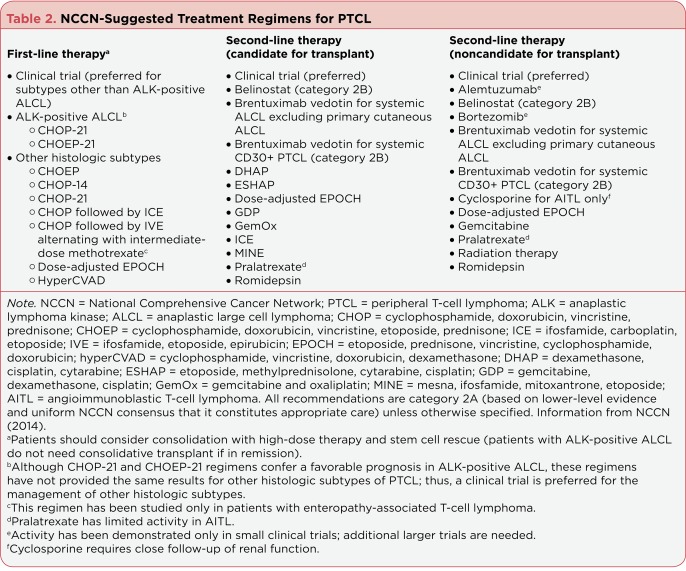
NCCN-Suggested Treatment Regimens for PTCL

With the exception of ALK-positive ALCL, common PTCL subtypes exhibit poor long-term survival, even with aggressive chemotherapy (Table 3; Vose et al., 2008). In a meta-analysis of 31 PTCL studies, the 5-year OS for patients treated with doxorubicin-containing regimens was 37% (Abouyabis et al., 2007). In a separate retrospective study of patients with PTCL, in which > 85% of patients with the most common subtypes received anthracycline-containing regimens, the median OS ranged from 1 to 5 years for subtypes other than ALK-positive ALCL (Vose et al., 2008). However, the better prognosis for ALK-positive ALCL may be related to age, because patients with this subtype tend to be younger than those with other subtypes (Foss et al., 2011; Savage et al., 2008). In an age-matched comparison of patients with ALK-negative vs. ALK-positive ALCL, there was no difference in failure-free survival or OS when considering either the group of patients aged 40 years or those aged < 40 years (Savage et al., 2008).

**Table 3:**
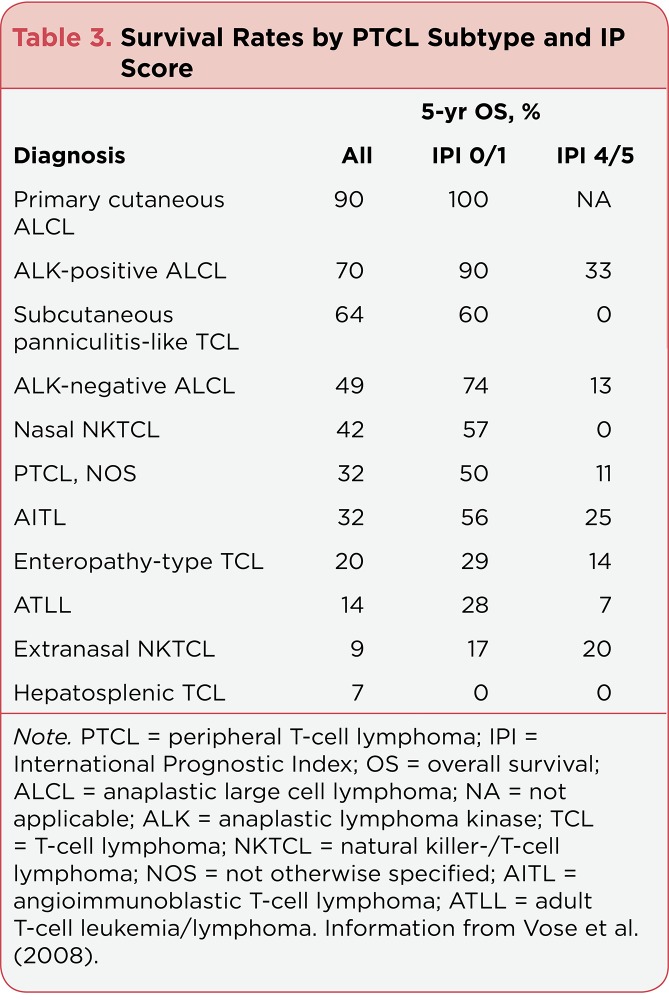
Survival Rates by PTCL Subtype and IP Score

The International Prognostic Index (IPI) was originally developed to determine the predictive risk factors of outcomes in patients with NHL (N = 2,031; International Non-Hodgkin’s Lymphoma Prognostic Factors Project, 1993). These criteria were applied to patients with PTCL and found to have prognostic value in this patient population (Vose et al., 2008). Significant risk factors for decreased survival include age > 60 years, stage III/IV disease, the presence of > 1 extranodal site, a performance status of 2, and a serum LDH level above normal (International Non-Hodgkin’s Lymphoma Prognostic Factors Project, 1993).

The sum of the number of risk factors present at diagnosis contributes to the patient’s relative risk of death. As seen in Table 3, the 5-year OS rate for patients with PTCL varied by subtype and by the number of IPI risk factors present (Vose et al., 2008). Generally, patients with ALCL had the best survival rates, with 5-year OS rates between 49% and 90% across all IPI scores (Vose et al., 2008).

A prognostic index specific to patients with PTCL, NOS (PIT) has also been described (Gallamini et al., 2004). By multivariate analysis, age > 60 years, Eastern Cooperative Oncology Group performance status 2, serum LDH level above normal, and bone marrow involvement of disease were independently predictive of survival, and the total number of the four risk factors present negatively impacted 5- and 10-year OS in a retrospective analysis of 385 patients from the Intergruppo Italiano Linfomi Lymphoma Registry (Gallamini et al., 2004).

## HDAC INHIBITORS AS ANTICANCER AGENTS

**Figure 2:**
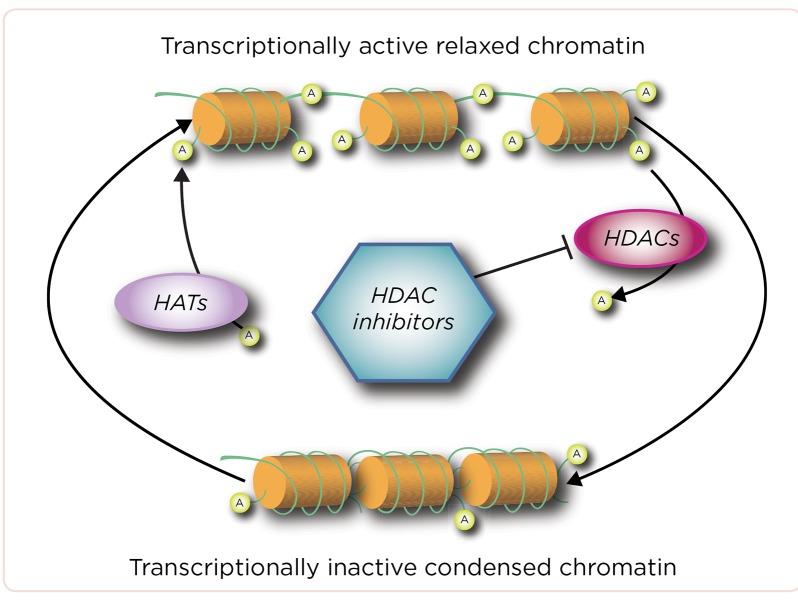
Figure 2. HDAC and HAT activity toward DNA. HAT = histone acetyltransferase; HDAC = histone deacetylase inhibitor. From Marsoni et al. (2008), New et al. (2012), Sajan & Hawkins (2012).

Histones are proteins that package DNA (see Figure 2 Marsoni, Damia, & Camboni, 2008; New, Olzscha, & La Thangue, 2012; Sajan & Hawkins (2012)). Enzymes called histone acetyltransferases (HATs) add acetyl groups to histones, which leads to an "open" chromatin conformation that results in DNA transcription and gene expression (Marsoni et al., 2008; New et al., 2012). HDAC inhibitors remove acetyl groups from histones, leading to a "closed" chromatin state that blocks transcription (Marsoni et al., 2008; New et al., 2012). The balance of histone acetylation and deacetylation drives normal cell growth and differentiation; however, aberrant HDAC activity has been noted during malignant transformation (Marsoni et al., 2008).

Furthermore, HATs and HDACs also regulate the acetylation of nonhistone proteins important for cell growth and differentiation (Marsoni et al., 2008). In cancer, nonhistone proteins—including those involved in proliferation and cell death—can be hypoacetylated by HDACs, indicating that HDACs are involved in multiple methods of dysregulation of cell function that lead to transformation (Marsoni et al., 2008).

There are 18 different HDACs known in humans, which are divided into classes I through IV. Most class I enzymes (HDAC-1, -2, -3, and -8) are expressed in the nucleus of cells throughout the body and are essential in cell survival and proliferation; other classes are more tissue-specific (Marsoni et al., 2008; New et al., 2012).

HDAC inhibitors prevent HDACs from removing acetyl groups, both allowing DNA to remain transcriptionally active and maintaining the acetylation of nonhistone proteins (Marsoni et al., 2008). Also, HDAC inhibitors can help to restore normal balance and inhibit cancer progression through several mechanisms, including inhibition of angiogenesis, activation of apoptosis, and progression to differentiation (Marsoni et al., 2008; New et al., 2012).

A number of different HDAC inhibitors have been investigated as anticancer agents (Khan & La Thangue, 2012; New et al., 2012). HDAC inhibitors vary in their chemical structures, their specificity for different classes of HDAC, and their potency (Bradner et al., 2010; Khan & La Thangue, 2012; New et al., 2012). Romidepsin is a structurally unique, potent, bicyclic class I HDAC inhibitor that is active at nanomolar concentrations (Bolden, Peart, & Johnstone, 2006; Bradner et al., 2010; Furumai et al., 2002; Tan, Cang, Ma, Petrillo, & Liu, 2010). Romidepsin has been approved for the treatment of relapsed/refractory PTCL and CTCL (Celgene Corporation, 2014), vorinostat (Zolinza) has been approved for the treatment of relapsed/refractory CTCL (Merck & Co., 2013), and belinostat has been approved for the treatment of relapsed/refractory PTCL (Spectrum Pharmaceuticals, Inc., 2014). Other HDAC inhibitors, including panobinostat, are also under investigation for use in T-cell lymphoma. However, equivalent efficacy in T-cell lymphoma is not a class effect of HDAC inhibitors.

Variations in the specificity and potency of HDAC inhibitors may result in differing levels of activity. For example, reported overall response rates (ORRs) in CTCL trials were 34% to 35% for romidepsin, 30% for vorinostat, 17% for panobinostat, and 14% for belinostat (Duvic et al., 2013; Olsen et al., 2007; Piekarz et al., 2009; Pohlman et al., 2009; Whittaker et al., 2010).

## ROMIDEPSIN

**Phase I Single-Agent Trials**

A phase I trial of 37 patients with a variety of tumor types investigated the maximum tolerated dose (MTD) of romidepsin (Sandor et al., 2002). The MTD was determined to be 17.8 mg/m² as a 4-hour intravenous (IV) infusion on days 1 and 5 of a 21-day cycle. The dose-limiting toxicities (DLTs) observed were nausea, vomiting, fatigue, and transient cytopenias. Because of the high rates of nausea and vomiting, patients were given antiemetics at doses of romidepsin 3.5 mg/m². Reversible electrocardiogram (ECG) changes were also noted at dose levels 3.5 mg/m²; however, there was no evidence of myocardial damage. This trial included 4 patients with T-cell lymphoma enrolled at the 12.7 or 17.8 mg/m² dose level, one of whom had PTCL (Piekarz et al., 2001). This patient had a CR to romidepsin after progressing on etoposide, prednisone, vincristine, cyclophosphamide, and doxorubicin (EPOCH) chemotherapy. A separate phase I study in patients with advanced cancer (n = 33) demonstrated similar toxicities (fatigue and thrombocytopenia as DLTs) and determined the MTD to be 13.3 mg/m² administered as a 4-hour IV infusion on days 1, 8, and 15 of a 28-day cycle (Marshall et al., 2002).

**Phase II Single-Agent Trials**

The FDA approval of romidepsin for the treatment of relapsed/refractory PTCL was primarily based on data from the pivotal GPI-06-0002 trial (Celgene Corporation, 2014; Coiffier et al., 2012). This trial enrolled patients with relapsed or refractory PTCL after at least one prior systemic therapy (Coiffier et al., 2012). Of 131 patients enrolled, 130 patients had histologically confirmed PTCL by central review. Patients received romidepsin 14 mg/m² as a 4-hour IV infusion on days 1, 8, and 15 of a 28-day cycle for up to 6 cycles and could continue on romidepsin beyond 6 cycles as long as they continued to experience benefit (stable disease or better) and tolerate the drug. Romidepsin dosing could be withheld or reduced to 10 mg/m² if required for the management of adverse events (AEs).

Separate response assessments were performed every two cycles by investigators and an independent review committee (IRC). The rigorous two-step IRC assessment (radiologic assessment followed by a broader clinical assessment) performed by expert radiologists and hematologic oncologists was used to determine the primary endpoint of CR/unconfirmed CR (CRu). The objective response rate by IRC was 25% (33 of 130), including 19 patients (15%) with CR/CRu (Coiffier et al., 2012; Coiffier et al., 2014).

Similar response rates and durations were seen across the most common subtypes of PTCL (PTCL, NOS; AITL; and ALK-negative ALCL; Coiffier et al., 2012; Coiffier et al., 2014). There were no significant differences in ORRs or rates of CR/CRu based on baseline characteristics such as gender, age (< 65 years vs. 65 years), IPI score (< 2 vs. 2), number of prior systemic therapies (< 3 vs. 3), prior stem cell transplant, prior monoclonal antibody therapy, prior nonantibody immunotherapy, or refractoriness to prior therapy (Coiffier et al., 2012). The median duration of objective response was 28 months (Coiffier et al., 2014), with the longest response ongoing at 56+ months (Foss et al., 2014). Of the 19 patients who achieved CR/CRu, 10 had responses that lasted 12 months or longer, and none of the baseline characteristics examined, including heavy pretreatment, response to prior therapy, or advanced disease, precluded long-term responses to romidepsin (Coiffier et al., 2014). Patients who achieved CR/CRu had substantially longer progression-free and overall survival compared with patients in other response categories, and patients who achieved partial response or stable disease for 90 days had similar long-term outcomes (Coiffier et al., 2014).

A similarly designed trial at the National Cancer Institute (NCI) that supported the PTCL indication enrolled 47 patients with relapsed/refractory PTCL—45 with confirmed trial eligibility (NCI-1312; Piekarz et al., 2011). Patients were treated with romidepsin as a 4-hour IV infusion on days 1, 8, and 15 of a 28-day cycle at a dose of 14 mg/m², amended from the initial dose of 18 mg/m² on days 1 and 5 of a 21-day cycle (Sandor et al., 2002, received by two patients) for improved tolerability. Doses could be withheld or reduced to 10.5 mg/m² or further to 8 mg/m² as necessary in the event of toxicity or escalated to 17.5 mg/m² in the absence of toxicity (Piekarz et al., 2011). The ORR (investigator assessed) was 38% (17 of 45 patients), including eight (18%) with a CR (Piekarz et al., 2011).

**Safety**

The most common side effects experienced in phase II trials of romidepsin for patients with relapsed/refractory PTCL were of hematologic or gastrointestinal origin (Celgene Corporation, 2014). In the GPI-06-0002 trial, side effects were manageable, and the most common drug-related AEs, nausea and asthenia/fatigue, were primarily grade 1/2 and did not result in treatment discontinuation (Coiffier et al., 2012). The most common grade 3 side effects were thrombocytopenia, neutropenia, infections (all types pooled), anemia, and asthenia/fatigue. The incidence of grade 3 AEs and treatment discontinuations was highest during the first two cycles of treatment (Foss et al., 2014).

In the NCI-1312 trial, the most common grade 3 side effects reported in cycle 1 were leukopenia, neutropenia, lymphopenia, thrombocytopenia, fatigue, anemia, and hyperuricemia (Piekarz et al., 2011).

The most common events leading to treatment discontinuation in phase II trials were thrombocytopenia, pneumonia, anemia, infection, and an increase in alanine aminotransferase levels (Celgene Corporation, 2014). Deaths within 30 days of romidepsin treatment occurring in the two phase II trials were most commonly due to progressive disease or infection/event occurring during infection (Coiffier et al., 2012; Piekarz et al., 2011).

Changes in ECG, including ST-segment and T-wave changes and/or QT prolongations, have been reported with romidepsin treatment (Coiffier et al., 2012; Piekarz et al., 2006; Piekarz et al., 2011; Sandor et al., 2002; Shah et al., 2006); however, these changes were not found to be clinically significant or associated with myocardial damage or impaired cardiac function (Noonan et al., 2013; Piekarz et al., 2006; Piekarz et al., 2011; Sandor et al., 2002).

An early analysis of a postmarketing study that examined changes from pre- and postantiemetic baselines reported that clinically insignificant changes in QTc were attributable to antiemetic premedication (Godfrey et al., 2011). Changes in ECG parameters, including QTc intervals, have been previously shown to be a class effect of antiemetic 5-hydroxytryptamine 3 receptor agonists, such as ondansetron (Keefe, 2002;  Navari & Koeller, 2003), which was commonly used as premedication with romidepsin (Godfrey et al., 2011). Patients treated with romidepsin may experience transient increases in heart rate, with no evidence of increased arrhythmia (Noonan et al., 2013).

**FDA Approval of Romidepsin in PTCL**

Romidepsin is indicated for the treatment of PTCL in patients who have received at least one prior therapy (Celgene Corporation, 2014). Romidepsin has no reported contraindications. The package insert warnings include cytopenias, infections, tumor lysis syndrome (TLS), and ECG changes. Women receiving romidepsin should avoid pregnancy because of potential fetal harm (Celgene Corporation, 2014). Romidepsin is administered as a 4-hour infusion at a dose of 14 mg/m² on days 1, 8, and 15 of each 28-day cycle as long as the patient continues to benefit from and tolerate the drug (Celgene Corporation, 2014).

Romidepsin is currently under clinical investigation in combination therapies for patients with PTCL. Romidepsin plus CHOP in a phase Ib/II trial of patients with newly diagnosed PTCL resulted in a preliminary ORR of 78%, including 66% with CR for the initial 14 evaluable patients (Dupuis et al., 2012). Significant but tolerable hematologic toxicity was observed in these patients. Furthermore, preclinical synergy of romidepsin and pralatrexate has been demonstrated (Jain et al., 2012). These and other ongoing trials may lead to additional PTCL indications for romidepsin.

## CONSIDERATIONS FOR ADVANCED PRACTITIONERS

**Assessment of Laboratory Data**

Prior to romidepsin administration, laboratory data should be assessed—including complete blood cell (CBC) counts and electrolytes. Hematologic abnormalities are common in patients with PTCL and may be exacerbated by prior myelosuppressive chemotherapy, thus CBCs should be performed regularly during treatment with romidepsin (Celgene Corporation, 2014; Coiffier et al., 2012).

The need for electrolyte supplementation is common in patients with T-cell lymphoma (Noonan et al., 2013), and hypomagnesemia and hypokalemia may be associated with ECG abnormalities including QT prolongation (Cabell et al., 2009; Piekarz et al., 2006). Thus, potassium and magnesium levels should be kept within the normal range to minimize the risk of QT prolongation (Celgene Corporation, 2014; Piekarz et al., 2011). The normal range for potassium is 3.5 to 5.0 mmol/L and for magnesium is 0.8 to 1.2 mmol/L (1.95 to 2.92 mg/dL; Kratz, Ferraro, Sluss, & Lewandrowski, 2004). In the phase II trials of romidepsin in PTCL, serum magnesium levels 0.85 mmol/L ( 2.06 mg/dL) and serum potassium levels 3.8 to 4.0 mmol/L were required (Coiffier et al., 2012; Piekarz et al., 2006; Piekarz et al., 2011). APs should confirm the targeted electrolyte levels for their institution.

**Administration Guidelines**

Romidepsin is administered by IV infusion over 4 hours and can be infused via a peripheral line; a central line is not preferred because of the high risk of infection (Frye et al., 2012). When an IV line is established, caution should be taken to avoid infections because infections (all types pooled) were among the most common side effects of romidepsin reported in clinical trials, and deaths relating to infection or sepsis have occurred (Celgene Corporation, 2014; Coiffier et al., 2012; Piekarz et al., 2011). Patients should be instructed to report fever, cough, shortness of breath with or without chest pain, burning on urination, flu-like symptoms, muscle aches, or worsening skin problems (Celgene Corporation, 2014).

On the basis of a population pharmacokinetic analysis, romidepsin drug exposure is not expected to be significantly influenced by mild hepatic or renal impairment. However, patients with moderate or severe hepatic impairment or end-stage renal disease should be treated with caution (Celgene Corporation, 2014). Cardiac monitoring should be considered in patients with congenital long QT syndrome, in patients with a history of significant cardiovascular disease, and in patients taking medicinal products that lead to significant QT prolongation (Celgene Corporation, 2014).

**Adverse Events and Management**

Patients receiving romidepsin may experience hematologic and nonhematologic AEs. Common AEs reported include thrombocytopenia, fatigue, nausea, vomiting, and changes in taste (Celgene Corporation, 2014). Hematologic abnormalities such as thrombocytopenia, neutropenia, and lymphopenia should be monitored during treatment with romidepsin (Celgene Corporation, 2014; Coiffier et al., 2012). Patients should be instructed to report fever or other signs of infection, significant fatigue, shortness of breath, or bleeding. If patients experience grade 3 or 4 neutropenia or thrombocytopenia, treatment with romidepsin should be delayed.

When the absolute neutrophil count returns to 1.5 × 10^9^/L and/or the platelet count returns to 75 × 10^9^/L or to baseline, romidepsin may be restarted at 14 mg/m². If patients experience grade 4 febrile neutropenia (fever of 38.5°C) or thrombocytopenia that requires platelet transfusion, treatment with romidepsin should be delayed. When cytopenia returns to grade 1 or baseline, the dose should be permanently reduced to 10 mg/m² (Celgene Corporation, 2014). Patients who experience changes in taste with romidepsin treatment should be referred to a dietitian.

Prophylactic antiemetics are recommended for all patients receiving romidepsin (Celgene Corporation, 2014) and may be required for the first 48 hours following treatment. Patients should be instructed to stay hydrated; increased oral hydration and light meals on treatment days can help manage nausea (Frye et al., 2012), and IV fluids may be helpful in some patients. Oral antiemetics may continue to be necessary for 24 to 48 hours after infusion in some patients (Frye et al., 2012).

There are no recommended antiemetics for use with romidepsin, and selection among them should be made by the individual providers. Based on the emetogenicity of romidepsin, we suggest that 5-hydroxytryptamine 3 (5-HT₃) receptor antagonist antiemetics are useful; however, institutional pharmacy formularies may dictate drug selection. Changes in ECG parameters, including QTc intervals, are a class effect of first-generation 5-HT₃ receptor antagonist antiemetics, such as the commonly used ondansetron (Keefe, 2002; Navari & Koeller, 2003). Granisetron may have less of an effect on the QT interval than ondansetron (Keefe, 2002), and the second-generation agent palonosetron did not significantly increase the QT interval (Yavas, Dogan, Yavas, Araz, & Ata, 2012; Gonullu, Demircan, Demirag, Erdem, & Yucel, 2012).

If patients experience nonhematologic toxicities (except alopecia), romidepsin should be delayed, and the dose may need to be reduced. If grade 3 or 4 toxicities recur after dose reduction, romidepsin should be discontinued (Celgene Corporation, 2014).

Tumor lysis syndrome has been reported in 2% of patients with stage III/IV PTCL treated with romidepsin (Celgene Corporation, 2014). This oncologic emergency occurs as a result of lysis of a large number of tumor cells, leading to the release of potassium, phosphate, and nucleic acids into the systemic circulation (Cairo, Coiffier, Reiter, Younes, & TLS Expert Panel, 2010). Development of this condition predisposes patients to a number of clinical toxicities, such as cardiac arrhythmia, seizures, renal insufficiency, or even sudden death (Cairo et al., 2010).

Patients with advanced-stage PTCL or a high tumor burden should be closely monitored for TLS (Celgene Corporation, 2014). Uric acid, potassium, or phosphate levels above the upper limit of normal or elevated 25% from baseline are biologic signs of TLS. Risk factors for TLS include renal dysfunction, elevated LDH level, and large tumor burden (Cairo et al., 2010).

**Documentation of Concurrent Medications**

There are a number of medications that may interact with romidepsin (Table 4). Advanced practitioners should routinely review the patients’ concurrent prescribed and over-the-counter medications and consult with pharmacists regarding potential interactions with romidepsin; their health-care provider should be notified of any changes. Particularly important are CYP3A4 inhibitors or inducers and those that cause QT prolongation (Celgene Corporation, 2014); patients should be monitored for toxicity related to increased romidepsin exposure when it is coadministered with strong CYP3A4 inhibitors, and coadministration with potent CYP3A4 inducers should be avoided. Caution should also be exercised in the case of medications that inhibit P-glycoprotein (Celgene Corporation, 2014).

**Table 4:**
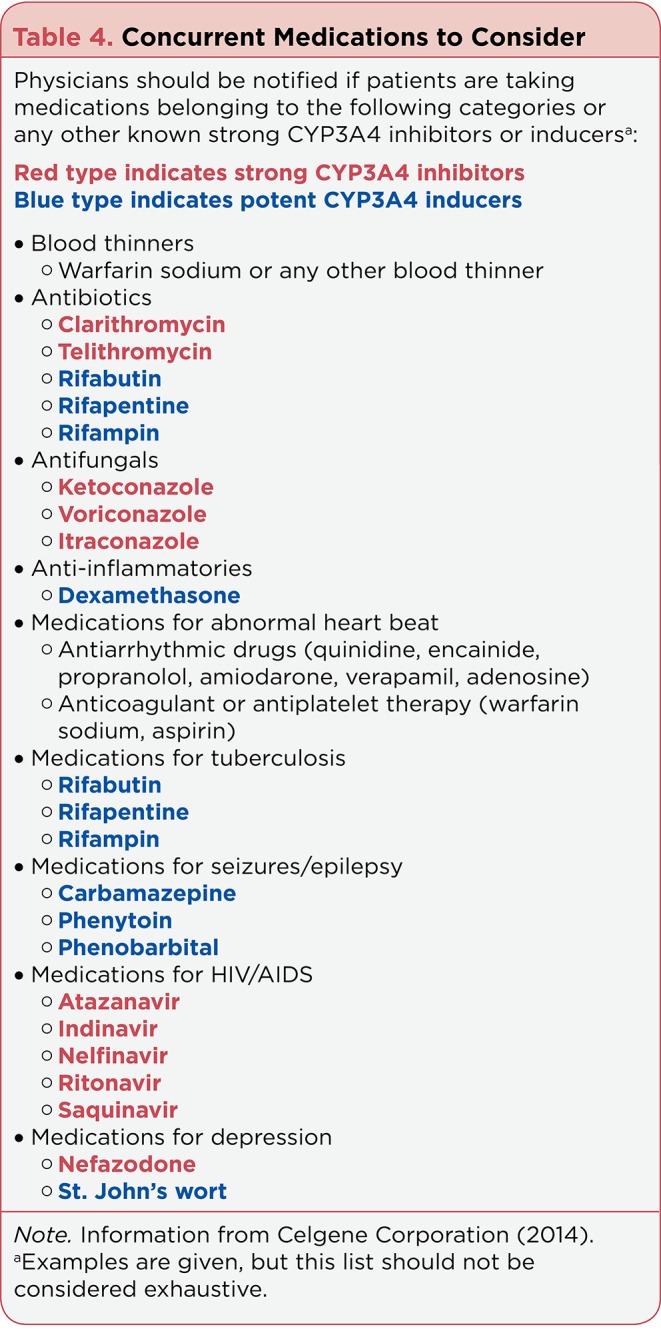
Concurrent Medications to Consider

Furthermore, concurrent use of romidepsin and warfarin may lead to an elevated international normalized ratio (INR) and potentiation of anticoagulation effects; careful monitoring of prothrombin time and INR is advised in patients taking romidepsin with warfarin or its derivatives (Celgene Corporation, 2014). In addition, romidepsin binds to estrogen receptors (Celgene Corporation, 2014) and may decrease the efficacy of estrogen-containing contraceptives; female patients should be cautioned to use alternate forms of contraception while being treated with romidepsin (Gloucester Pharmaceuticals, 2009).

Patients with cancer often have many different health-care providers. Patients must be educated to inform all of their providers that they are taking romidepsin, and an assessment of potential interactions should be undertaken before any new medication is prescribed.

## CONCLUSION

Patients with PTCL typically have a poor prognosis and often experience inadequate responses despite aggressive first-line chemotherapy. Romidepsin is a single-agent therapy that can lead to durable responses in patients with relapsed/refractory PTCL. Advanced practitioners are in a key position to identify and manage treatment- specific complications and also play a critical role in educating patients on side effects, reportable signs and symptoms, and medication interactions. Therefore, APs must have a clear understanding of this new therapy.

**Acknowledgments**

Ms. Barbarotta and Ms. Hurley met the International Committee for Medical Journal Editors criteria for authorship, were fully involved in development of all drafts of this manuscript, and assume responsibility for the direction and content. The authors thank the following individuals for their editorial assistance with production of the manuscript: Beth Burke, PhD, and Stacey Rose, PhD (MediTech Media) for editorial services; these services were funded by Celgene.
